# Impact of Subspecialty Fellowship Training on Research Productivity Among Academic Plastic Surgery Faculty in the United States

**Published:** 2015-11-18

**Authors:** Aditya Sood, Paul J. Therattil, Stella Chung, Edward S. Lee

**Affiliations:** Division of Plastic Surgery, Department of Surgery, Rutgers University–New Jersey Medical School, Newark

**Keywords:** *h*-index, fellowship training, academic productivity, research productivity, academic promotion

## Abstract

**Purpose:** The impact of subspecialty fellowship training on research productivity among academic plastic surgeons is unknown. The authors’ aim of this study was to (1) describe the current fellowship representation in academic plastic surgery and (2) evaluate the relationship between *h-*index and subspecialty fellowship training by experience and type. **Methods:** Academic plastic surgery faculty (*N* = 590) were identified through an Internet-based search of all ACGME-accredited integrated and combined residency programs. Research output was measured by *h-*index from the Scopus database as well as a number of peer-reviewed publications. The Kruskal-Wallis test, with a subsequent Mann-Whitney *U* test, was used for statistical analysis to determine correlations. **Results:** In the United States, 72% (*n* = 426) of academic plastic surgeons had trained in 1 or more subspecialty fellowship program. Within this cohort, the largest group had completed multiple fellowships (28%), followed by hand (23%), craniofacial (22%), microsurgery (15%), research (8%), cosmetic (3%), burn (2%), and wound healing (0.5%). Higher *h-*indices correlated with a research fellowship (12.5; *P* < .01) and multiple fellowships (10.4; *P* < .01). Craniofacial-trained plastic surgeons demonstrated the next highest *h-*index (9.8), followed by no fellowship (8.4), microsurgery (8.3), hand (7.7), cosmetic (5.2), and burn (5.1). **Conclusion:** Plastic surgeons with a research fellowship or at least 2 subspecialty fellowships had increased academic productivity compared with their colleagues. Craniofacial-trained physicians also demonstrated a higher marker for academic productivity than multiple other specialties. In this study, we show that the type and number of fellowships influence the *h-*index and further identification of such variables may help improve academic mentorship and productivity within academic plastic surgery.

Plastic surgeons in academic practice are crucial in preparing the future surgeons in our field. Identifying the processes that correlate with the pursuit of higher academic achievement may help improve academic mentorship.

Advancement within academic plastic surgery is multifactorial, with research output being one of the most heavily examined aspects in determining productivity.^1^^-^5 There are several frequently used metrics to assess research productivity, including total number of publications, “significant” number of publications, and number of citations by other authors in peer-reviewed literature.^6^^,^^7^ Although easily quantifiable, these metrics do little to measure the overall impact of research productivity.

The *h-*index is a powerful tool that can help evaluate both the quantity and quality of an individual author's contributions in academia. This tool was first proposed in 2005 by physicist Hirsch^7^ at the University of California, San Diego, in which he defined an *h-*index as the number of *h* articles that have been cited at least *h* times. For example, if an academic plastic surgeon has an *h-*index of 10, it means that he or she has 10 publications, with at least 10 citations each. This metric is not skewed by the total number of publications as the number of citations is accounted for. This allows for the *h-*index to be a potentially more accurate measure of research productivity than pure quantitative metrics.

There are several online medical literature databases available for calculating this score, including Scopus and Google Scholar.^8^ Although results of *h*-indices between the 2 aforementioned sources may vary, previous analysis evaluating the *h*-indices between academic neurosurgeons found a high degree of correlation in the calculated scores between Scopus and Google Scholar.^2^

There have been previous works published in plastic surgery analyzing the influence of training institution on *h-*index, as well as gender disparities and academic productivity.^1^^,^^9^ There has been no previous analysis in plastic surgery evaluating whether this objective quantification of research impact is affected by fellowship training. It is the authors’ hypothesis that training in multiple subspecialty plastic surgery fellowships will have a positive impact on academic productivity. To study this, we examined the training attributes of academic plastic surgery faculty at accredited combined and integrated plastic surgery training institutions. Our aims were to (1) evaluate the relationship between *h-*index and fellowship training by experience and type and (2) to describe the current fellowship representation in academic plastic surgery to assess whether there are any correlations to be drawn.

## MATERIALS AND METHODS

A list of plastic surgery residency programs was accessed from the American Medical Association Fellowship and Residency Interactive Database. This included departments with either or both integrated and traditional track residencies. Independent fellowship training institutions were excluded. Faculty lists were accessed from individual training program Web sites, and a total of 590 faculty members were included in this analysis after exclusion of nonacademic clinical faculty, instructors, nonphysician research faculty, adjunct, and part-time faculty. An institutional review board approval was not obtained for this retrospective study.

Academic rank and fellowship information of faculty members was obtained from department Web sites, physicians’ personal Web sites, and an online third-party physician database such as the US News and Health Report (health.usnews.com). The *h-*index was calculated for each faculty member using the Scopus database (www.scopus.com; Elsevier, Philadelphia, Pa). All data were collected in August 2014.

Surgeons who trained in more than 1 fellowship were classified in the “multiple fellowships” category. Surgeons who trained in general plastic surgery fellowship, those who trained in general surgery–related fellowships (such as surgical oncology), and those with no available fellowship training information were included in the “no-fellowship” category.

Subspecialty fellowships of interest included hand, craniofacial, microsurgery, cosmetic, research, wound healing, and burn. “Hand fellowship” included hand and microsurgery, hand, wrist, and peripheral nerve, and pediatric hand training. “Craniofacial fellowship” included craniofacial and pediatrics, pediatric craniomaxillofacial, orthognathic, and cleft and craniofacial training. “Microsurgery fellowship” included breast and microsurgery, head and neck oncology and microsurgery, and reconstructive surgery training. “Cosmetic fellowship” included aesthetic and minimally invasive, breast reconstruction, and aesthetic surgery training.

Statistical analyses were conducted using the Kruskal-Wallis test, with a subsequent Mann-Whitney *U* test, using Microsoft Excel, version 2007, software. Thresholds for significance were set at *P* < .05.

## RESULTS

A total of 590 academic plastic surgeons from 80 residency programs were identified and evaluated after our exclusion criteria. Thirty-one percent (*n* = 183) were professors, 24% (*n* = 140) were associate professors, and 45% (*n* = 267) were assistant professors. Among them were 81 chiefs and chairpersons. Seventeen percent (*n* = 101) were female and 83% (*n* = 489) were male.

**Figure 1:**
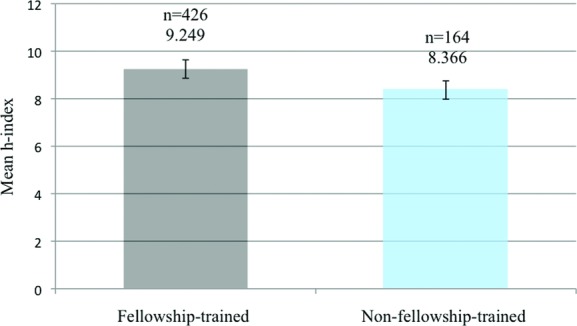
Mean *h*-indices of academic plastic surgeons organized by fellowship experience. *n* represent sample size, error bars represent standard error of the mean. *P* value calculated from the Kruskal-Wallis and Mann-Whitney *U* tests. *P* = .11.

**Figure 2:**
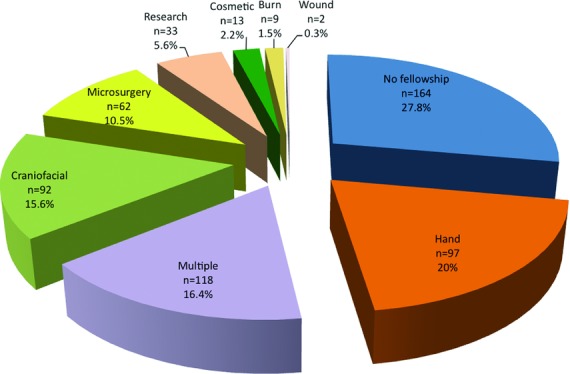
Subspecialty composition of plastic and reconstructive surgeons studied (*n* = 590).

**Figure 3:**
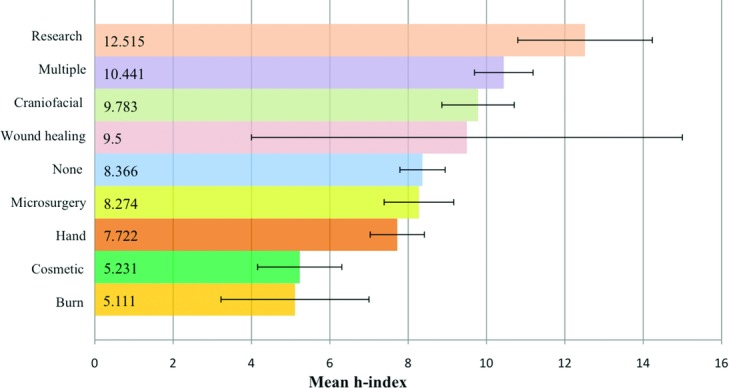
Mean *h*-indices of the academic plastic surgeons organized by plastic subspecialty. Error bars represent standard error of the mean. *P* < .05.

Seventy-two percent (*n* = 426) of academic surgeons have trained in at least 1 plastic subspecialty fellowship (vs 28% or *n* = 164 with no subspecialty training). The mean *h-*index in the fellowship group versus the “no-fellowship” group was 9.3 versus 8.4 (*P* = .11; Fig 1). Within the fellowship-trained cohort, multiple fellowships (*n* = 118) were the most common, followed by hand (*n* = 97), craniofacial (*n* = 92), microsurgery (*n* = 62), research (*n* = 33), cosmetic (*n* = 13), burn (*n* = 9), and wound healing (*n* = 2) (Fig 2). Total mean *h-*index was 9.10. Research fellowship-trained surgeons had a mean *h-*index value of 12.5, the highest value among all other subspecialties (Kruskal-Wallis test and Mann-Whitney *U* test, *P* < .05). The second highest mean *h-*index of 10.4 was observed in the multiple fellowships group (*P* < .05; Fig 3).

**Figure 4:**
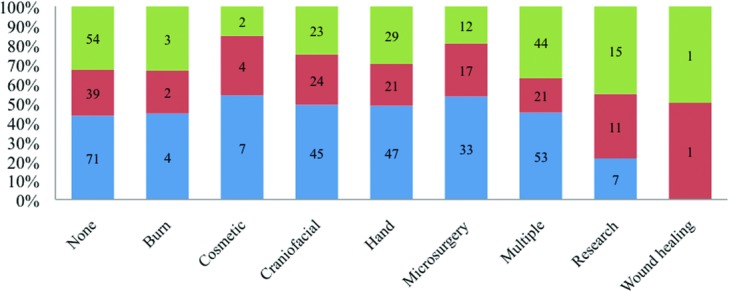
Academic rank breakdown of plastic surgeons. Green bar represent proportion of professor faculty, red bar represents proportion of associate professor faculty, blue bar represents proportion of assistant professor faculty, and *n* represents absolute number.

**Figure 5:**
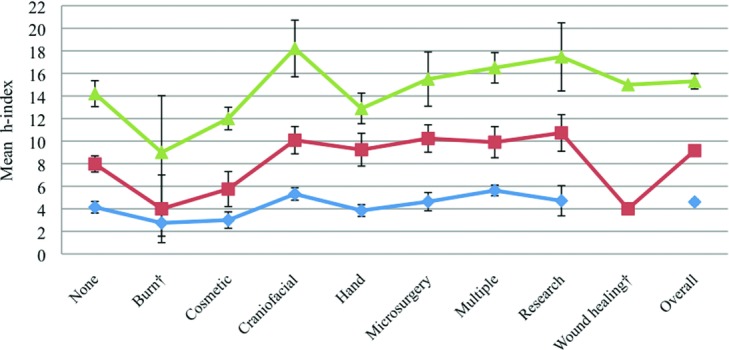
Mean *h*-index of plastic surgeons stratified by academic rank and fellowship training. Green dots represent professor faculty, red bars represent associate professor faculty, blue dots represent assistant professor faculty, error bars represent standard error of the mean. *P* value between senior and junior ranks calculated from the Kruskal-Wallis test with a subsequent Mann-Whitney *U* test. *P > .05.

Surgeons were further distributed by academic rank. Fellowship-trained surgeons comprised 70% (129/183) of professors, 72% (101/140) of associate professors, and 73% (196/267) of assistant professors. Wound healing and research fellowships had the largest proportions of professorship and associate professorship, whereas more than half of cosmetic and microsurgery fellowship-trained surgeons were assistant professors (Fig 4). Professors had significantly higher mean *h*-indices than the junior academic ranks across all fellowship categories except in burn and wound healing (Kruskal-Wallis test and Mann-Whitney *U* test, *P* < .05; Fig 5).

**Figure 6:**
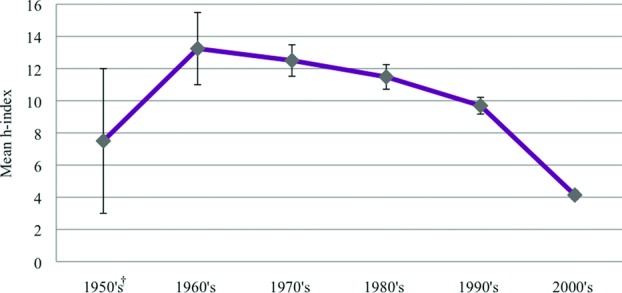
Mean *h*-index of plastic surgeons distributed by year of medical school graduation. Error bars represent standard error of the mean. *P* value between senior and junior graduates calculated from the Kruskal-Wallis test with a subsequent Mann-Whitney *U* test. *P > 0.05.

There was a positive correlation between *h-*index growth and time since medical school graduation (*P* > .05 for 1950s, *P* < .05 for 1960-2000s; Fig 6). Twelve surgeons were excluded from this analysis because of the lack of available information regarding their medical school graduation year.

## DISCUSSION

This study examined 590 academic plastic surgeons from 80 academic departments. We studied the current distribution of fellowship training among academic surgeons and whether fellowship training had an effect on their academic productivity. In the United States, approximately three-fourths (72%) of plastic surgery academic faculty members have undergone at least 1 subspecialty fellowship training program (Fig 1). This is comparable with the proportion of fellowship-trained practitioners in the fields of otolaryngology (77%), ophthalmology (83%), and neurosurgery (66%).^10^^-^12 This phenomenon can be explained by the ability to obtain competitive professorship with postsecondary training experience. The most common training obtained by academic plastic surgeons was multiple fellowships (Fig 2). Surgeons interested in academia may pursue multiple fellowships to gain further knowledge and skills in more than 1 subspecialty. In reverse, surgeons with multiple fellowships training acquire diverse clinical and surgical proficiency, which may provide them with more opportunities to enter academia.

The *h-*index has been used as an important marker of scholarly impact in various fields of medicine.^2^^,^^12^^-^29 Current metrics of academic productivity are total number of citations, total number of publications, and citations per publication, and *h-*index is a single statistic that incorporates all 3 factors.^9^ Previous publications have identified and proved the value of *h-*index as a predictive role in future academic productivity, research grant funding, and academic promotion.^7^^,^^9^ There are limitations of using *h-*index as a measurement of academic productivity. *h-*index is may be inflated by the following: negatively or disproved citations; favored citations of famous authors over lesser well-known individuals; and self-citations.^9^^,^^10^^,^^30^^-^33 To minimize bias, we have excluded self-citations in the *h-*index calculation process.

The mean *h-*index of all academic surgeons was 9.1. There was no significant difference in the mean *h-*index between the fellowship-trained and non–fellowship-trained groups, although it approached a trend (*P* = .11; Fig 1). Similar findings have been reported from the fields of urology and neurosurgery.^11^^,^^34^ In these surgical specialties, *h-*index may not be affected by fellowship training itself but by gender, type of residency program, posttraining academic position, or institution the surgeon is affiliated with. Our results differed from a previously published study of academic plastic surgeons that reported a significantly higher mean *h-*index in the fellowship-trained group.^1^ The discrepancy is most likely due to excluding faculty from independent plastic surgery institutions in our analysis. A significantly higher *h-*index in fellowship-trained physicians has also been reported from the fields of ophthalmology and otolaryngology.^1^^,^^10^ Factors that are likely to be associated with a higher *h-*index were male gender, faculty from frequently represented training programs, no private practice affiliation, and academic rank.^1^^,^^9^ The variation noted among different surgical specialties may be impacted by several factors such as the number of surgeons, research emphasis within a specific field, and inherent bias within the data collection process. Therefore, comparison within a given field rather than between specialties is currently recommended.^10^

Despite the lack of significant correlation between fellowship training and *h-*index, a trend was observed upon stratification by fellowship type. Research fellowship-trained surgeons comprised only 8% of the faculty, yet they had the highest mean *h-*index (Fig 3). Both personal interest and emphasis on research and publishing in the research group are evident factors leading to a high *h-*index. Research fellowship provides time and resources for a surgeon to focus on innovative research. This is possible in clinical fellowships as well; however, practice is often busier with increased clinical duties required to generate revenue and sustain a practice.^1^ Furthermore, research fellowships are not restricted to a particular subspecialty or time frame. While clinical fellowships are typically 1- or 2-year programs, postresidency research fellowships can last for any number of years, thus allowing the surgeons to complete experiments and/or trials to publish their findings. The second highest *h-*index was seen in the multiple fellowships-trained cohort. Many surgeons in this category also have research background that increases their scholarly impact.

We further distributed fellowship-trained surgeons by academic position. We found that 70% of professors, 72% of associate professors, and 73% of assistant professors have completed fellowship training. Similar proportions across all ranks indicate that postsecondary training experience has been traditionally important and is not a recent phenomenon. Professors had the highest mean *h-*index of 15.3 and continued to maintain a significantly higher mean *h-*index than their juniors across all fellowships, with exception to burn and wound healing (Fig 5). Our findings are consistent with previously published studies showing higher *h-*indices associated with higher academic ranks.^9^^,^^12^^,^^34^

Burn and research fellowship-trained surgeons had the greatest proportion of both professor and associate professor faculty members, whereas cosmetic and microsurgery fellowship-trained surgeons had the greatest proportion of assistant professors (Fig 4). This may indicate a current shift of plastic surgery practice to cosmetic surgery and microsurgery as well as increased academic opportunities for surgeons specializing in these fields. Alternatively, one may speculate that cosmetic surgery and microsurgery are newer fellowships that were less available to senior plastic surgeons at the time of their residency completion.

One of the criticisms of using *h-*index as a static measurement of academic productivity is its lack of control for the time a publication may take into account.^6^^,^^7^ We did indeed observe that the duration of medical practice as determined by the year of medical school graduation had an overall positive effect on *h-*index growth (Fig 6). The graduation year of plastic surgeons ranged from 1950s to 2000s. The highest *h-*index was observed in the 1960s group rather than the 1950s group. We believe that the small sample size (*n* = 2) in the 1950s group is the cause of deviation from an otherwise linear trend. Inflation and deflation of *h-*index must be noted. Some surgeons have obtained degrees such as PhD and DMD after medical school. The additional years in school have been unaccounted for, due to the limited information available online, leading to inflation of actual years of medical practice. In addition, *h-*index of senior faculty may be deflated since Scopus includes only cited publications before 1996. Overall, the time of practice versus *h-*index trend is relevant and meaningful in illustrating that time is needed for *h-*index growth. With time, practitioners gain in-depth knowledge and become more clinically proficient, increasing their scholarly impact.

There are other limitations to this study. Eloy et al^10^ have extensively discussed the limitations in using Scopus as a single database for *h-*index calculation. Several other databases exist with inherent variations, such as Google Scholar, but studies show that one particular database is unlikely to result in inaccurate conclusions.^10^^,^^15^ In addition, a surgeon's academic productivity is a multifaceted dimension. It not only comprises research work but also includes clinical expertise and teaching.^9^ *h-*index does not encompass all of the mentioned factors but is frequently used as an index to quantify a physician's scholarly impact.^9^ Plastic surgery is unique in that surgeons from various residencies such as otolaryngology, oral and maxillofacial surgery, and orthopedic surgery participate in plastic surgery fellowship training. Therefore, fellowship population and data may be more variable than other surgical fields.

In this study, we showed the current status of fellowship-trained academic plastic surgeons. We examined their academic research output distributed by type of fellowship, academic rank, gender, and time. A surgeon's scholarly impact is an important tool in academic job decisions such as hiring and promotion, and it also has a role in predicting grant funding.^35^ Differences in *h*-indices among fellowship-trained faculty should encourage more mentoring and educational resources in underrepresented fellowships. As time proved to be an important factor in one's scholarly impact, we expect that junior faculty will see an eventual increase in their academic productivity. The same principle can be applied to fellowship categories associated with lower *h*-indices.

## CONCLUSION

*h-*index offers a quantifiable and objective alternative to other metrics when evaluating research productivity and academic advancement. This metric appears to be highly associated with subspecialty fellowship training within the field of plastic surgery in the United States. Plastic surgery faculty with a research fellowship or at least 2 subspecialty fellowships demonstrated increased academic productivity compared with their colleagues (*P* < .05). Craniofacial-trained physicians also demonstrated a higher *h-*index than multiple other trainings. In this study, we show that the type and number of fellowships influence the *h-*index and further identification of such variables may help improve academic mentorship and productivity within academic plastic surgery.
